# 484. Vaginal *Candida albicans:* High Frequency of *In-vitro* Fluconazole Resistance in a Select Clinical Population.

**DOI:** 10.1093/ofid/ofac492.542

**Published:** 2022-12-15

**Authors:** Pranjal Agrawal, Golsa M Yazdy, Khalil G Ghanem, Victoria L Handa, Jack D Sobel, Sean Zhang, Susan Tuddenham

**Affiliations:** Johns Hopkins University School of Medicine, Baltimore, Maryland; Johns Hopkins University, Baltimore, Maryland; Johns Hopkins University, Baltimore, Maryland; Johns Hopkins School of Medicine, Baltimore, Maryland; Wayne State University School of Medicine, West Bloomfield, Michigan; Johns Hopkins Hospital, Baltimore, Maryland; Johns Hopkins, Baltimore, Maryland

## Abstract

**Background:**

*In vitro* fluconazole resistance in vaginal *C. albicans* has rarely been reported in the U.S. Little is known about the characteristics of patients who demonstrate fluconazole resistance vs. sensitivity, or how likely resistant strains are to persist over time. Hence we sought to describe patterns of fluconazole resistance in clinician ordered vaginal cultures positive for *C. albicans* at our center. We compared patient characteristics between those with fluconazole sensitive versus resistant isolates.

**Methods:**

We conducted a chart review of patients with clinician ordered vaginal cultures positive for *C. albicans* undergoing fluconazole susceptibility testing in our medical center’s clinical mycology lab from January 2017 to April 2021. Patient characteristics were compared using chi-square and ANOVA tests and correlated to fluconazole resistance with logistic regression models.

**Results:**

Of N=92 patients with vaginal *C. albicans*, 3.3% were sensitive dose-dependent (SDD: minimal inhibitory concentration (MIC) =4) to fluconazole, and 30.4% were resistant (R: MIC > =8). Amongst those with at least 2 previous episodes of vulvovaginal candidiasis (VVC) in the past 6 months (N=45), 50% had R and 6.5% had SDD isolates. Compared to those with sensitive (S: MIC < =2) isolates, patients with SDD or R were younger and more likely to have the following characteristics: Black race, bacterial vaginosis (BV) in the past year, high or multi-dose azole treatment, fluconazole suppressive therapy in the past 6 months, and more VVC and BV episodes in the previous 6 months (Table 1). Of N=10 patients who had a follow-up culture with initial R isolates, 28.6% had a sensitive *C. albicans* vaginal isolate.

**Figure:**
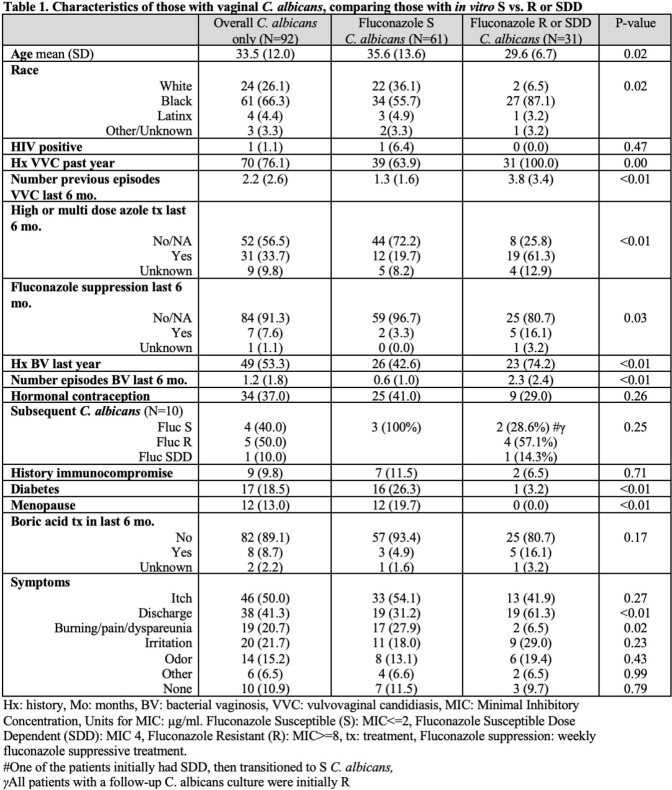

**Conclusion:**

The frequency of *C. albicans* vaginal isolates with *in vitro* resistance to fluconazole in this study was high. Our findings highlight the importance of obtaining cultures and sensitivity testing in patients with recurrent or refractory VVC. However, resistant vaginal strains may not necessarily persist over time. Future, larger studies on those with recurrent VVC are needed to verify factors associated with fluconazole resistance, how frequently azole-resistant strains persist over time, and how well *in vitro* resistance correlates to *in vivo* treatment response.

**Disclosures:**

**Susan Tuddenham, MD, MPH**, Luca Biologics: Advisor/Consultant|Medscape/WebMD: Honoraria|UpToDate: Royalties.

